# Multiple Sclerosis: Immunopathology and Treatment Update

**DOI:** 10.3390/brainsci7070078

**Published:** 2017-07-07

**Authors:** Narges Dargahi, Maria Katsara, Theodore Tselios, Maria-Eleni Androutsou, Maximilian de Courten, John Matsoukas, Vasso Apostolopoulos

**Affiliations:** 1 Centre for Chronic Disease, College of Health and Biomedicine, Victoria University, Melbourne VIC 3030, Australia; narges.dargahi@live.vu.edu.au (N.D.); Maximilian.deCourten@vu.edu.au (M.d.C.); 2 Medical Department, Novartis (Hellas) SACI, Metamorphosis, Athens 14452, Greece; maria.katsara@novartis.com; 3 Department of Chemistry, University of Patras, Rio, Patras 26500, Greece; ttselios@upatras.gr; 4 Vianex S.A., Metamorphosis, Attikis, Athens 14451, Greece; AndroutsouM@vianex.gr; 5 ELDrug S.A., Patras Science Park, Platani, Patras 26504, Greece; imats1953@gmail.com

**Keywords:** multiple sclerosis, immunotherapy, drug delivery, vaccine

## Abstract

The treatment of multiple sclerosis (MS) has changed over the last 20 years. All immunotherapeutic drugs target relapsing remitting MS (RRMS) and it still remains a medical challenge in MS to develop a treatment for progressive forms. The most common injectable disease-modifying therapies in RRMS include β-interferons 1a or 1b and glatiramer acetate. However, one of the major challenges of injectable disease-modifying therapies has been poor treatment adherence with approximately 50% of patients discontinuing the therapy within the first year. Herein, we go back to the basics to understand the immunopathophysiology of MS to gain insights in the development of new improved drug treatments. We present current disease-modifying therapies (interferons, glatiramer acetate, dimethyl fumarate, teriflunomide, fingolimod, mitoxantrone), humanized monoclonal antibodies (natalizumab, ofatumumab, ocrelizumab, alemtuzumab, daclizumab) and emerging immune modulating approaches (stem cells, DNA vaccines, nanoparticles, altered peptide ligands) for the treatment of MS.

## 1. Introduction

In the early 1900s, only a few cases of multiple sclerosis (MS) were reported, which quickly became a common occurrence for admission to neurological wards. Today, MS accounts over 2.5 million affected individuals with an estimated cost of US$2–3 billion per annum [1]. The distribution of MS varies according to geographic location. For example, the further north or south from the equator the higher the prevalence of MS; countries that lie on the equator have extremely low prevalence compared to Scotland, Norway, and Canada. The prevalence of MS has increased progressively over time with 30/100,000 diagnosed in 2008 to 33/100,000 diagnosed in 2013 globally. In fact, in a Norwegian cohort over 53 years (1961–2014), the prevalence increased from 20 to 203/100,000 and the incidence increased from 1.9 to 8/100,000 [2]. It is possible that the increase in prevalence is due to improved diagnostic procedures and reporting and changes in lifestyle (lack of vitamin D and increased smoking) [1]. MS is commonly diagnosed between 20 years and 40 years of age although it can affect younger and older individuals [3], and most commonly affects those with a genetic predisposition (major histocompatibility complex (MHC) class II phenotype, human leukocyte antigen (HLA)-DR2 and HLA-DR4 most commonly affected). In fact, the incidence of MS is increased 10-fold in monozygotic twins as compared to siblings of patients with MS [4,5,6]. In addition, viral infections can trigger disease where parts of the virus mimics that of the myelin sheath [7]. Although usually not life-shortening, MS is a chronic neurological disease often interfering with life and career plans of an individual [8].

MS is categorized into 4 distinct types, primarily based on its clinical course, which are characterized by increasing severity: (a) Relapsing/remitting MS (RRMS), the most common form, affecting 85% of all MS patients which involves relapses followed by remission; (b) secondary progressive MS (SPMS), which develops over time following diagnosis of RRMS; (c) primary progressive MS (PPMS) affecting 8–10% of patients, noted as gradual continuous neurologic deterioration; and (d) progressive relapsing MS (PRMS) the least common form (<5%), which is similar to PPMS but with overlapping relapses [9,10,11]. MS leads to a wide range of symptoms with various severity involving different parts of the body. MS diagnosis is mainly clinically based however, magnetic resonance imaging (MRI) assists in diagnosis [12]. As such, examination of the cerebrospinal fluid (CSF) and visual induced potentials with MRI can assist in confirming the clinical suspicion of MS [12,13]. MS symptoms and disease progression are varied, with some individuals experiencing little disability while most (up to 60%) require a wheelchair 20 years from diagnosis [9].

Although treatments against MS are able to decrease the relapse rate in RRMS, the prevention of long-term effects remains a problem; medications for progressive forms of MS are also limited in their efficacy. Hence, new improved drugs are required to effectively treat MS. One of the major pathophysiological mechanisms of MS involves autoreactive T cells, primarily T helper (Th)-1 CD4^+^ T cells and Th17 cells leading to cytokine secretion and activation of an inflammatory cascade resulting in demyelination within the brain and spinal cord and axonal damage; autoreactive antibodies cannot be discounted. Indeed, MS is generally known as a chronic autoimmune disorder of the central nervous system (CNS) [14,15]. MS causes breakdown of the blood brain barrier (BBB) leading to migration of immune cells (macrophages, T cells, B cells) and secretion of pro-inflammatory cytokines and chemokines [16] which induces inflammation, formation of sclerotic plaques (lesions), demyelination and neurodegeneration [17]. MS lesions may form in any location of the CNS white matter or in grey matter, often leading to physical disability and sometimes, decline in cognitive ability [16,18]. It is therefore, conceivable to target immune cells and their products in order to prevent tissue damage by modulating inflammation [9,19] while reducing potential side effects such as global immunosuppression [6,19,20]. The major constituents of the myelin sheath in which autoreactive T cells and antibodies recognize, include, myelin basic protein (MBP), myelin oligodendrocyte glycoprotein (MOG) and proteolipid protein (PLP).

## 2. Immunopathophysiology of MS

The brain has primarily been considered to be an organ which is highly immune-advantaged, although a number of studies have challenged this [6]. In the last 10 years an important shift has surfaced in MS research, suggesting that MS is not just a disease of the immune system, but equally involves factors contributed by the CNS [21,22]. Immune cells residing in the CNS get activated following damage to CNS tissue; notably microglial cells whereby they upregulate MHC class I and II molecules and cell surface co-stimulatory molecules and secrete cytokines and chemokines, paving entry for T (CD4 and CD8) cells, B cells, monocytes, macrophages and dendritic (DC)-like cells into CNS lesions [6]. Infiltrating immune cells secrete pro-inflammatory cytokines, nitric oxide, and matrix metalloproteinases [23,24], leading to destruction of the myelin sheath.

**Figure 1:**
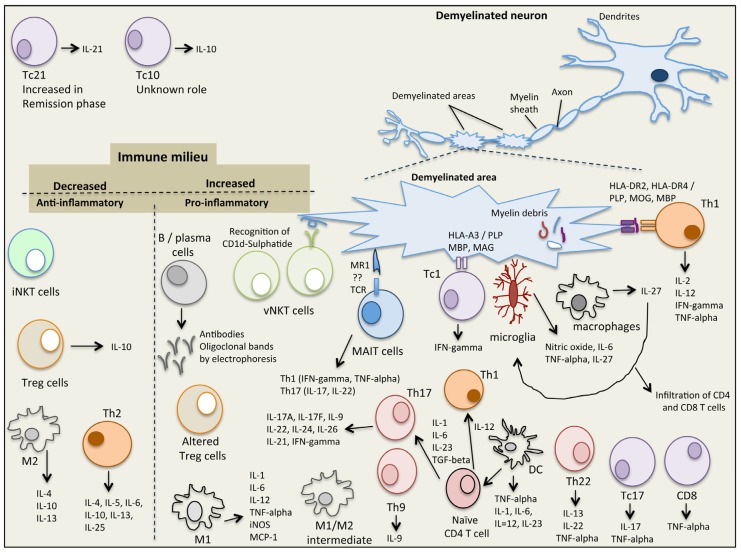
The immunological complexity of the immune/cytokine network in multiple sclerosis.

It has been generally accepted that chronic inflammation is the hallmark of neurodegenerative diseases, such as MS, Alzheimer’s disease and Parkinson’s disease [6,7]. Myelin-reactive auto-T cells cross the BBB [19] and their migration into the CNS consequently initiates an inflammatory cascade followed by demyelination of the CNS and axonal damage. These cells reside in the perivenous demyelinating lesions which generate distinct inflammatory demyelinated plaques situated within the white matter [25]. MS lesions appear in the white matter inside the visual neuron, basal ganglia, brain stem and spinal cord [26]. White matter cells transmit neural signals from grey matter, where information is gathered, and transferred to the rest of the body [25,27]. MS involves 2 main steps, (i) myelin sheath damage resulting in formation of lesions in the CNS and (ii) inflammation, which together destroy the neuron tissue [25,28]. In MS, damage of oligodendrocytes and destruction of myelin sheath leads to breakdown of the nerve axon and loss of neuronal function [28]. Demyelination increases the inflammatory activation processes leading to damage of BBB and stimulation of macrophage activation and oxidative stress pathways [29]. The white matter lesions include myelin breakdown together with infiltration of monocytes, B cells, T cells and DC [30]. Microglia and macrophages are the main innate immune cells present in MS lesions where they either act together with T and B cells, or directly cause neuroinflammatory tissue damage [31]. Cells involved in the inflammatory process include those that are both in the innate and adaptive immune systems and are described below (Figure 1).

### 2.1. Natural Killer T (NKT) Cells

NKT cells share properties of both T cells and NK cells and recognize glycolipid antigens presented in complex with the MHC class I-like molecule, CD1d. Two subsets of NKT cells have been identified (type I, invariant NKT (iNKT) cells and type II, variant NKT (vNKT) cells) and are implicated in the pathogenesis of MS in humans and in the murine model of MS, experimental autoimmune encephalomyelitis (EME). iNKT cells express cell surface markers characteristic of activated or memory T cells (CD25, CD44, CD69) with the majority being CD4^+^ as well as markers characteristic of NK cells (NK1.1 or CD161, Ly49). Following activation of iNKT cells (via binding to α-GalCer-CD1d complex) an array of cytokines is secreted that are associated with both pro- and anti-inflammatory immune responses and play a role in both innate and acquired immunity. As such, iNKT cells, (i) secrete interleukin (IL)-4 and IL-13 which stimulate CD4^+^ T cells to differentiate into anti-inflammatory Th2 cells (IL-4, IL-10 producers) which inhibit Th17, Th1, CD8^+^ T cells in the CNS; (ii) secrete IL-2 and tumor growth factor (TGF)-beta which stimulate the production of T regulatory (Treg) cells (IL-10, TGF-beta producers) which inhibit Th17, Th1 and CD8^+^ T cells in the CNS; and (iii) secrete IL-4, IL-10, IL-13, interferon (IFN)-gamma and GM-CSF which activate suppressive myeloid derived suppressor cells (MDCs), DC and macrophages which in turn secrete IL-10 to activate Treg cells and suppress Th17, Th1 and CD8^+^ T cells in the CNS [32]. Due to the pleiotropic properties of iNKT cells, they play a role in protecting the host against pathogens, tumors, autoimmunity and are involved in tissue rejection, ischemia reperfusion injury and obesity related diabetes [32]; deficiency or dysfunction of iNKT cells has been shown to be linked to the development of autoimmune diseases. Indeed, iNKT cell numbers are decreased in patients with MS [32] and are restored in patients in remission [33]. Analysis of iNKT cells in MS patients in remission showed a Th2 cytokine profile, suggesting an immunoregulatory effect of iNKT cells in MS [34]. Similarly, in the EAE mouse model, protection of EAE development is associated with high levels of iNKT cells and suppression of Th1 and Th17 cells [35]. Interestingly, injections of α-Galactosylceramide (α-GalCer), and analogues thereof, have potent activities in protecting mice against, cancer, infections, inflammatory conditions and autoimmune disorders. Hence, it is possible to develop iNKT cell based modulating therapies against MS [36,37]. Like iNKT cells, variant NKT (vNKT) cells also share properties of both T cells (CD4^+^) and NK cells (NK1.1) and recognize β-linked glycolipid antigens in complex with CD1d. They are less common in mice compared to iNKT cells but are more abundant in humans. Of interest, vNKT cells recognize the self-glycolipid, sulphatide, which is abundantly expressed within the myelin sheath suggesting a role in MS although not yet established [38]. Likewise, vNKT cells recognizing sulphatide self-myelin ligand are present in high levels in mice with EAE suggesting their role in disease progression [38].

### 2.2. Mucosal-Associated Invariant T (MAIT) Cells

MAIT cells are a subset of T cells of the innate immune system to defend against microbial infections. They are present in the liver, lungs, mucosa and blood and make up to 25% of CD8 T cells in healthy individuals; they also support adaptive immune responses in that they have a memory like phenotype [39]. The MHC class I-like molecule, MRI, presents microbial antigens and vitamin B metabolites to MAIT cells, leading to their activation [39,40]. However, MAIT cells have also been implicated in autoimmune diseases such as MS, inflammatory bowel disease and rheumatoid arthritis where they are often noted at the site of autoimmune attack. Recently, it was reported that in MS, MAIT cells are highly present at the sites of demyelination and secrete pro-inflammatory Th1 cytokines (IFN-gamma and TNF-alpha) and activate Th17 cells (IL-17 and IL-22 cytokines) [22]; the major cytokines in the pathogenesis of chronic inflammatory and autoimmune diseases. In addition, MAIT cell have been noted in white matter inflammatory lesions [41] as well as transcription over expression of MR1 in MS lesions. Conversely, it has been reported that MAIT cells are decreased in blood of patients with RRMS [42]. It is not clear whether MAIT cells exert a protective or a non-protective role, thus a better understanding of how MAIT cells are involved in MS and of their interactions would aid in a better understanding of the pathogenesis of MS and development of therapeutic strategies.

### 2.3. Regulatory T Cells (Tregs)

Regulatory T cells (Tregs; originally known as suppressor T cells) are a subset of CD4^+^ T cells that modulate immunity, maintain tolerance against self-antigens and prevent autoimmunity. Tregs are primarily characterized as Foxp3^+^CD25^+^CD4^+^ and are anti-inflammatory (secrete IL-10). One of the first evidence of the role of Treg cells in MS was in mouse EAE models, where adoptive transfer of Treg cells from control mice into MOG or PLP induced EAE mice prevented the onset and progression of EAE [43,44]. Adoptive transfer of Treg cells recovering from EAE into MOG-induced active EAE mice resulted in resolution of EAE [45]. In addition, induction of Treg cells by estradiol or by monocytes under glatiramer acetate treatment reduced clinical signs of MOG-EAE [46,47]. Furthermore, injection anti-CD28 monoclonal antibody in Lewis rats results in Treg cell expansion and reduction in EAE disease severity [48]. Interestingly, injection of anti-CD25 monoclonal antibody, which blocks the effects of Treg cells into C57BL/6 mice increased susceptibility to EAE induction [45]. In patients with MS however, the frequency of Foxp3^+^CD25^+^CD4^+^ Treg cells does not differ to those in healthy individuals, although the function of such cells are impaired (maturation and migration) [49]. In addition, mRNA and protein levels of Foxp3 are impaired in Treg cells of patients with MS especially in RRMS and are normalized during SPMS [49]. Hence, impaired functionality of Treg cells is primarily observed in the early stages of MS but not in their chronic stage, suggesting a causative role [50]. Further studies of Treg cells in MS may aid in the understanding for why tolerance against self-antigens is broken, leading to disease. However, it is not clear whether the impaired function of Treg cells is a direct cause of MS or whether such impairment is a general outcome for all autoimmune disorders.

### 2.4. Macrophages and Microglia

Macrophages are divided into M1 or M2 based on their pro- or anti-inflammatory cytokine secretion phenotype [51]. M1 macrophage phenotype of mice (F4/80^+^CD11b^+^CD11c^+^iNOS^+^) and human (CD40^+^CD86^+^CD64^+^CD32^+^) is induced in the presence of interferon (IFN)-gamma and/or toll-like receptor (TLR) ligands such as lipopolysaccharide (LPS). M1 macrophages are pro-inflammatory and primarily secrete IL-1, IL-6, IL-12, TNF-alpha, iNOS and MCP-1 [51]. In general, they stimulate adaptive immune responses. The M2 macrophage phenotype of mice (F4/80^+^CD11c^−^CD301^+^Arg1^+^CD206^+^) and humans (CD163^+^CD206^+^) is induced in the presence of IL-4, IL-10, IL-13 and Arg1 that blocks iNOS activity [51]. M2 macrophages are anti-inflammatory and primarily secrete IL-1 receptor antagonist, IL-4, IL-10, transforming growth factor (TGF)-beta1. Macrophages play a crucial role in the pathogenesis of MS. In fact, in active demyelinating and early re-myelinating lesions, macrophages are highly present compared to inactive, demyelinated or late re-myelinated lesions [52]. However, a distinction of M1 vs M2 macrophages in human brain tissues is not so clear, with both M1 macrophages and an intermediate subtype (M1/M2, CD40^+^CD206^+^) being present [53]. Like macrophages, microglia cells are divided into M1- and M2-polarized microglia cells. M1 microglia cells are pro-inflammatory and express CD40, CD74, CD86 and CCR7, whereas, M2 microglia cells are anti-inflammatory and express mannose receptor (CD206) and CCL22. In MS brain lesions however, like macrophages, an intermediate microglia phenotype is present expressing CD40, CD74, CD86 and CCL22 but not CD206 markers [54]. Interestingly, in an EAE model it was shown that suppression of CCL22 decreased M1 macrophage accumulation in the CNS, thus therapies designed to suppress CCL22 have the potential to decrease demyelination and progression of disease. In addition, in mice M1 microglia cells have been found to switch to M2 microglia cells during remyelination, hence M2 polarization is necessary for efficient remyelination [55]. Indeed, fasudil (a selective Rho kinase inhibitor), injected into EAE bearing mice shifted M1 to M2 macrophages and ameliorated the clinical severity of EAE [56].

### 2.5. T Helper Cells

CD4 T cells or T helper (Th) cells, recognize short 9–17 amino acid peptides presented on the surface of antigen presenting cells (APC) in complex with MHC class II. CD4 T cells differentiate into distinct Th cells depending on the cytokine secretion profiles [57]. (i) Th1 cells are pro-inflammatory and produce high levels of IL-2, IL-12, TNF-alpha and IFN-gamma; (ii) Th2 cells are anti-inflammatory and secrete IL-4, IL-5, IL-6, IL-10, IL-13, IL-25; (iii) Th17 cells are pro-inflammatory and secrete high levels of IL-17A, IL-17F, IL-21, IL-22, IL-24, IL-26 and low levels of IL-9 and IFN-gamma; (iv) Th22 cells which are a combination of Th1, Th2, Th17 phenotype and secrete IL-13, IL-22 and TNF-alpha and (v) the newest addition to the Th subset, Th9, was identified for its potent secretion of IL-9. Th1, Th9, Th17 cells are key contributors to MS by increasing inflammation within the milieu of the myelin site.

Th1 cells and their pro-inflammatory cytokine products are present in high levels within the demyelinating axon and CNS lesions of humans and in MOG, PLP or MBP induced EAE in mice. Th1 cells recognize MOG, PLP and MBP peptide epitopes presented in the context of MHC class II, HLA-DRB1*1501 (HLA-DR2, HLA-DR15) and HLA-DRB1*04 (HLA-DR4) alleles. As a result CD4 T cells become activated, cross the blood brain barrier and induce CNS autoimmunity. Some drug therapeutics target the MHC class II-peptide-T cell receptor (TCR) complex in an attempt to modulate or divert Th1 responses to therapeutic Th2 responses. Indeed, it was recently shown that dimethyl fumarate (DMF) injection in RRMS patients reduced Th1, Th17 and CD8 T cells and increased Th2 cells; this resulted in high levels of IL-4 and decreased levels of IFN-gamma and IL-17 [58]. In addition, we have shown that mannan conjugation of self-MBP, PLP or MOG native peptides or altered peptide ligands, are able to divert Th1 responses to Th2 responses in human PBMC from MS patients, in immunized mouse spleen cells and are able to ameliorate EAE in mice [59,60,61,62,63,64,65,66,67,68,69,70,71,72,73]. The role of Th9 cells in MS is not as clear although in mice, IL-9 and Th9 cells induce EAE and inflammation and IL-9 knockout mice are protected from developing EAE [74]. Th17 cells play a crucial role in the pathogenesis of MS in both mice and humans by inducing an inflammatory milieu. In fact, IL-17A is present at high levels in CNS lesions, cerebrospinal fluid and in the serum of patients with MS [75]. Th17 cells express high levels of CCR6 which binds to the ligand CCL20 on vascular endothelial cells, enabling their entry through the blood brain barrier where they secrete pro-inflammatory cytokines including IL-17A. In addition, IL-17 interferes with the remyelination process. Of interest, anti-IL-17A humanized neutralizing monoclonal antibody (AIN457 or Secukinumab) injected in patients with MS showed reduction of lesions compared to placebo-treated control subjects [75]. In addition, Th22 cells are highly present in the peripheral blood and cerebral spinal fluid of patients with active RRMS [76], and IL-22 mRNA and Th22 cells are increased in relapsing MS compared to remitting MS patients [77]. Furthermore, Th22 cells specifically recognize MBP and are resistant to IFN-beta therapy [76].

IL-27, a member of the IL-6/IL-12 cytokine family, is secreted by macrophages, dendritic cells and microglia cells, with pleiotropic roles in immunomodulation being either pro- or anti-inflammatory. IL-27 also stimulates or inhibits T cell differentiation. Th1 cells are induced by IL-27 whereas Th2, Th17 and Treg cells are inhibited by IL-27. In addition, Tr1 cells a specialized subset of T cells which secrete IL-10 are induced in the presence of IL-27 [78]. In 40 patients with RRMS, circulating plasma IL-27 levels were significantly higher compared to healthy control subjects [79]. Likewise, IL-27 and IL-27R are elevated in post-mortem MS brain lesions compared to non-MS control brains. Macrophages and microglia were identified to be the source of IL-27 and triggering infiltration of CD4 and CD8 T cells [80]. In addition, the effects of IL-27 on microglia cells showed that nitric oxide, TNF-alpha and IL-6 were secreted, promoting Th1 polarization, suggestive that IL-27 enhances microglia neuroinflammation [81]. Hence, suppressing IL-27 may be a strategy to modulate inflammatory responses in patients with MS.

### 2.6. CD8 T Cells

Classical CD8 T cells or cytotoxic T cells (Tc1 cells), recognize short antigenic 7-9-mer peptide epitopes presented on the surface of APC in complex with MHC class I. In MS there is a genetic association with HLA-A3 [82]; HLA-A2 has been shown to reduce the risk of MS in individuals that also express MHC class II, HLA-DRB1*1501. The antigen specificity of CD8 Tc1 cells isolated from patients with MS, has been suggested to be against MOG, MBP and PLP with cytolytic activity against neuronal cells in vitro [83] although their pathogenic role in MS is still not clear. More recently other subsets of CD8 T cells have been identified and are grouped into different subsets based on their cytokine profile. In as such, classical Tc1 cells secrete IFN-gamma, Tc2 secrete IL-4, Tc10 secrete IL-10, Tc17 secrete IL-17, Tc21 secrete IL-21, Tc22 secrete IL-22 and another subset is characterized by secreting TNF-alpha. In MS, regardless of the stage and activity of disease CD8 T cells are noted in high numbers, much higher than CD4 T cells at a ratio of 10:1 CD8:CD4 T cells. MHC class I is highly expressed within MS lesions and astrocytes, oligodendrocytes, neurons in addition to the classical APC, DCs and macrophages. In fact, CD8 T cells are found in great abundance within CNS tissues and cerebrospinal fluid of patients with MS. CD8 T cells present in both acute and chronic MS lesions secrete high levels of IL-17 (classed as, Tc17 CD8 T cells) [84]. Tc17 cells secrete IL-17 and TNF-alpha and low IFN-gamma and are negative for granzyme B, perforin and cytolytic activity unlike the classical CD8 Tc1 cells. In peripheral blood of patients with SPMS and RRMS elevated levels of Tc1 and Tc17 cells are noted as well as a high percentage of TNF-alpha secreting CD8 T cells [85]; Tc21 cells are increased in the remission phase of RRMS compared to SPMS. In addition, higher levels of CD8^+^IFN-gamma^+^TNF-alpha^+^IL-17^+^ T cells in the relapsing phase of RRMS compared to remission phase, SPMS and controls [85]. It is clear that CD8 T cells contribute to the pathogenesis of MS, and it is important to understand how such cells escape T cell tolerance and induce CNS autoimmunity in order to design and develop new therapeutics against MS.

### 2.7. B Cells

Although there is a presence of T cells in MS plaques, B cells also contribute to the pathogenesis of MS where they secrete autoantibodies and cytokines and being APC they activate T cells. In patients with MS the presence of oligoclonal bands (OCB) in cerebrospinal fluid and brain parenchyma is a consistent finding in over 95% of patients. OCB is a product of clonally expanded B cells and IgG synthesis. In MS plaques plasma cells are noted in large numbers where antigen uptake, processing and presentation takes place as well as synthesis of IgG. Interestingly, over 50 antibodies isolated from cerebrospinal fluid from patients with MS did not react to MBP, PLP or MOG [86] but some groups reporting that they bind to intracellular proteins such as, MKNK1/2, FAM84A, AKAP12A and glial potassium channel KIR4.1, or, against intracellular lipid determinants [87,88]. Moreover, anti-MOG autoantibodies is a hallmark of childhood MS as well as in some patients with neuromyelitis optical spectrum disorder. It is clear, that abnormal activation of B cells within the CNS of patients with MS, suggests that B cells play a role in the pathophysiology of the disease. Further studies are required to ascertain whether B cell depletion is able to restore immune function and hence, be used as a therapeutic target against MS.

### 2.8. Dendritic Cells

DC are professional APC which process and present antigenic peptide epitopes on their surface in complex with MHC class I or class II, resulting in CD4 or CD8 T cell stimulation respectively. Even though MS is generally associated with predominant auto-reactive T cells, emerging evidence indicates that DCs play an important role in the pathophysiology of MS, primarily due to their T cell activating and cytokine secreting properties. Following activation of DCs in the periphery, T cells specific to myelin epitopes are activated inducing pro-inflammatory cytokines aiding their entry through the BBB into the CNS. In the CNS resident APC and T cells are further activated leading to demyelination and motor deficits. In patients with MS, DCs are abundantly present within inflamed lesions, cerebrospinal fluid and in the circulation and produce high levels of TNF-alpha, IFN-gamma and IL-6 [89]. In addition, the expression of co-stimulatory molecules, CD40 and CD80 on DCs are increased in RRMS and SPMS patients, suggesting an activated pro-inflammatory state of DCs, hence their contributing role in the pathogenesis of MS.

### 2.9. Myeloid Derived Suppressor Cells

Myeloid-derived suppressor cells (MDSC) are myeloid progenitors, the same lineage to that of macrophages, DC and neutrophils. However, MDSC have strong immunosuppressive properties rather than immune-stimulatory properties as noted with macrophages, DC and neutrophils [90]. Their major role is in tumor development and chronic inflammation having immune suppressive effects [90]. As such, it was recently shown following MBP_1–11_ peptide immunization in mice, that MDSCs were increased adopting a suppressive phenotype, inhibiting the activation of CD4^+^ T cells via arginase-1 and inducible nitric oxide synthase; such approach inhibited the development of EAE in mice [91]. In addition, MDSC secrete inhibitory enzyme indoleamine 2,3-dioxygenase and Th2 cytokine, IL-10 [92]. It is not clear whether the number of MDSCs are reduced or whether their functionality is altered in patients with MS, leading to the failure of MDSCs to suppress autoimmune T cells, as a result of disease progression. The use of ex vivo cultured MDSCs could be a viable strategy to develop new improved treatments against MS.

## 3. Current Drug Therapies for Multiple Sclerosis

**Figure 2:**
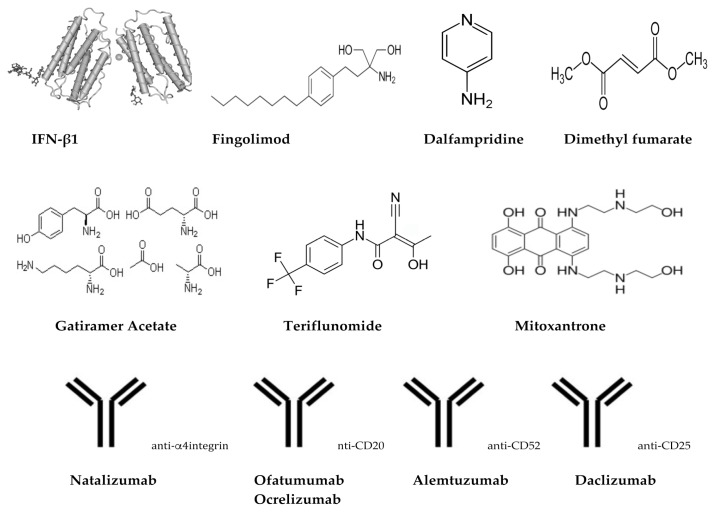
Chemical/schematic structures of treatments/drugs for MS.

The majority of the treatments for MS are long term mainly suppressing the immune system however, such immune-suppressants pose increased risks for infections and cancer [27]. Alternative treatment options involve disease-modifying therapies such as, interferons, glatiramer acetate, monoclonal antibodies and sphingosine-1-phosphate receptor modulators (Table 1, Figure 2). These therapies have dramatically reduced the number of attacks and decreased disease progression. In fact, interferons are effective in the early relapsing phases of MS but not in the advanced phases of the disease [27]. Ultimately, induction of tolerance against self-antigens and re-establishing immune homeostasis can effectively “cure” the disease; such strategies have been the focus of recent research.

### 3.1. Treatment of MS Relapses

Patients with MS who present with a relapse are generally treated with corticosteroids intravenously, plasma exchange or adrenocorticotropic hormone injections [50,93]. Although effective in reducing the duration of the relapse and patients recovery faster there are no long-term neuroprotective benefits [27,94,95,96,97].

### 3.2. Long-Term Treatment of MS with Disease-Modifying Agents

The treatment of MS has been a challenge with treatment options being limited mainly to corticosteroids, the potent alkylating agent cyclophosphamide and potent immunosuppressant methotrexate (Table 1, Figure 2). However, with the advent of immunomodulatory drugs in mid-1990s, a big shift was carried to treatment options for the first time [50]. The first disease-modifying drug for RRMS, interferon beta-1(IFNβ-1) was the primary key breakthrough for the treatment of MS [98,99]. Disease-modifying agents intend to modify the course of the disease rather than improving symptoms.

Until the approval of the first oral treatment in 2010 [11], all MS treatments consisted of either intramuscular or subcutaneous injectable drugs. To date, 13 FDA approved disease-modifying drugs are available for RRMS, and several more agents are in different developmental stages [9,11,65,66,69]. In the last 20 years there has been an evolving trend in novel treatments for MS and the global progress of therapies for MS has been quite promising. In general treatments consist of Ampyra^®^, Aubagio^®^, Avonex^®^, Betaseron^®^, Copaxone^®^, Extavia^®^, Gilenya^®^, Lemtrada^®^, Novantrone^®^, Plegridy^®^, Rebif^®^, Tecfidera^®^ and Tysabri^®^ [100]. Such treatment options consist of alemtuzumab (depletes lymphocytes), daclizumab (blocks the cytokine receptor IL-2), dimethylfumarate (combines features of immunomodulatory and immunosuppressive actions), fingolimod (modulates the sphingosine-receptor system), natalizumab (inhibits the migration of lymphocytes) and teriflunomide (inhibits activated T and B cells) [9,27,50]. Examples of current interferons include, Schering AG’s Betaferon/Betaseron (IFNβ-1b), Biogen’s Avonex (IFNβ-1a) and Serono/Pfizer’s Rebif (IFNβ-1a). In addition, immune modulating agents include, Teva’s Copaxone^®^ (copolymer glatiramer acetate), Amgen/Serono’s (Novantrone^®^; mitoxantrone), azathioprine, cyclophosphamide (Endoxan^®^) and Natalizumab^®^ an a_4_-integrin antagonist [101,102,103]. Disease-modifying agents have commonly been shown to reduce the rate of relapses, reduce MRI lesions and stabilize or delay MS disability. The key therapeutic features of disease-modifying drugs are their anti-inflammatory effects in the relapsing phase of MS, although demyelination leading to chronic disability still remains a major hurdle [27,104,105,106]. Some studies, however, have shown that early intervention of disease-modifying drugs to patients with RRMS can reduce acute disability or death [27,107,108,109,110].

In general, disease-modifying drugs main action is by suppressing or altering the immune system. Hence, based on this theory that MS is, at least in part, a result of altered or abnormal immune response that results in attack of the myelin sheath. Current available drugs and their actions are described below (Table 1, Figure 2).

#### 3.2.1. Interferons (Avonex^®^, Biogen, Cambridge, MA, USA; Betaseron^®^, Bayer, Leverkusen, Germany; Extavia^®^, Novartis Pharma AG, Basel, Switzerland; Rebif^®^, EMD Serono Inc., Darmstadt, Germany; Plegridy^®^, Biogen, Cambridge, MA, USA)

Interferon (IFN) type 1 consist of a group of IFNs (IFN-α, -β, -ε, -κ, -τ, -δ, -ζ, -ω, -ν) which help regulate the immune system. IFN-β is primarily produced by fibroblasts but other cells such as NK cells, B cells, T cells, macrophages also secrete IFN-β. IFN-β has anti-viral and anti-tumor activity as well as being effective in reducing the relapse rate in patients with MS [106]. The mechanism by which IFN-β acts, is that it balances the expression of pro- and anti-inflammatory cytokines in the brain and decreases the number of inflammatory cells crossing the blood brain barrier. As a consequence, there is decreased inflammation of neurons, increases nerve growth factors and improves neuronal survival. Moreover, IFN-β reduces Th17 population and IL-17 cytokine which are known to be involved in the immunopathophysiology of MS [111]. IFN-β injection subcutaneously or intramuscularly to patients with RRMS aims to decrease the relapse rate, duration and severity, however, there is lack of efficacy to long-term disability. Avonex was approved in 1996, the first FDA approved treatment for RRMS. To date there are 3 approaches using IFN-β; IFN-β1a low dosage (Avonex^®^), IFN-β1a (Rebif^®^) high dosage, and, IFN-β1b (Betaseron^®^, Extavia^®^) high dosage. Furthermore, pegIFN-β-1a (Plegridy^®^) has polyethylene glycol linked to IFN-β-1a allowing it to be active for longer in the body, hence fewer injections are required compared to Avonex^®^, Rebif^®^, Betaseron^®^ and Extavia^®^. The first large scale human clinical trial in patients with RRMS using IFN-β was published in 1993 and showed that relapse rates were reduced by 34% in high dose IFN-β1b and by 8% in lower dose compared to placebo group and severity of relapses were also reduced [112]. Subsequent 5 year follow-up data showed that IFN-β1a and IFN-β1b decreased lesions up to 30% and reduced the formation of new lesions up to 50%, however, the study failed to show any reduction in disability progression in patients [113]. IFNs have no direct neuroprotective effects, however, through their direct effect on CD4^+^Th1 cells and altering their profile results in decreased demyelination of neurons, which prevents further neuronal damage [114]. Despite the impact of IFN-β in disease progression in patients with RRMS there are limitations in their use, with side effects ranging from local body aches, skin reactions (swelling, redness), fever, myalgia, flu-like symptoms to more serious side effects such as suicidal thoughts, hallucinations, seizures and heart and liver problems [9]. As a result, many patients have stopped treatment and overall the benefit of using IFNs is relatively small.

#### 3.2.2. Glatiramer Acetate (Copaxone^®^, Inc., Petah Tikva, Israel)

Glatiramer acetate (GA) is a synthetic 4-mer peptide (l-glutamic acid, lysine, alanine, and tyrosine) mimic of MBP, which competes with short antigenic MBP peptides in complex with MHC class II. Initially, GA was designed to induce EAE but instead it suppressed EAE, which was quickly translated into human trials with MS in order to prevent disease progression, as it bound to MHC class II and inhibited the activation of encephalitogenic T cells [115,116,117,118]. GA diverts Th1 cells to Th2 cells that suppress inflammatory responses and activate Tregs in the periphery [119]. In patients, GA significantly reduced disease symptoms and development of new lesions by up to 30% in RRMS, although it showed no improvement in long-term efficacy on progression of disability [120]. GA injection in patients results in side effects ranging from minor (fever, chills) to more serious (cardiovascular, digestive, muscular, respiratory issues).

#### 3.2.3. Dimethyl Fumarate (Tecfidera^®^, Biogen, Cambridge, MA, USA)

Dimethyl fumarate (BG-12) is a methyl ester of fumaric acid that modulates immune responses and was approved by the FDA in 2013. BG-12 was shown in phase III clinical trials to reduce relapse rate and increase the time to disability progression in patients with RRMS [121]. BG-12 reduces the migration of inflammatory cells through the blood brain barrier and activates nuclear factor erythroid 2-related factor (Nrf2) [122]. Nrf2 regulates anti-oxidative proteins that protect cells against oxidative damage and inflammation. In fact, BG-12 protects neuronal cells from oxidative stress by increasing glutathione levels and suppressing pro-inflammatory cytokines from splenocytes in vitro [123]. Side effects of BG-12 include diarrhea, abdominal pain, nausea, abnormal liver enzymes and decreased lymphocyte counts.

#### 3.2.4. Teriflunomide (Aubagio^®^, Sanofi Genzyme, Cambridge, MA, USA)

Teriflunomide is an active metabolite of leflunomide (an immunosuppressive disease-modifying drug used for rheumatoid arthritis) which inhibits the enzyme dihydroorotate dehydrogenase [124] and inhibits the proliferation of B and T cells. In addition, teriflunomide exerts anti-inflammatory properties by inhibiting IFN-gamma producing T cells while IL-4 and IL-10 producing T cells are unaffected [125]. In MS, oral administration of teriflunomide reduced relapse rates, MS lesions and decreased disability progression [126,127,128,129,130,131]. Moreover, permanent discontinuation due to side effects was substantially less common in MS patients who received teriflunomide compared to IFN-β-1a. Side effects include, reduced white blood cell count, alopecia, hepatic effects, nausea, diarrhea, numbness in hand and feet, allergic reactions, breathing issues and increased blood pressure. Teriflunomide was approved by the FDA in 2012 and by EMA in 2013 for use in patients with RRMS.

#### 3.2.5. Fingolimod (Gilenya^®^, FTY720, Novartis Pharma AG, Basel, Switzerland)

Fingolimod was granted FDA approval in 2010 and was the first oral therapy (0.5 mg once daily) available for patients with relapsing forms of MS. Fingolimod is a sphingosine 1-phosphate (S1P) receptor modulator, which acts as a super agonist of S1P receptor causing receptor internalization and leading to reduced infiltration of potentially auto-reactive lymphocytes into the CNS, and as such, they remain localized in the lymph nodes [132,133,134]. In addition, a secondary beneficial effects of fingolimod is that it targets SIP receptors on glia cells in the CNS, activating signaling pathways within the CNS [132,135]. Based on Phase III human clinical trials in patients with RRMS (TRANSFORMS, FREEDOMS and FREEDOMS II), fingolimod was more effective compared to first line treatment IFNβ-1a and placebo, in reducing the frequency of flare-ups (clinical exacerbations), disability progression, MRI outcome measures, including brain volume loss and was associated with clearly identified adverse events [103,136,137]. More than 180,000 patients have been treated with fingolimod in clinical trials and post-marketing settings globally, and the total patient exposure now exceeds 395,000 patient-years. Side effects include bradycardia (within 6 h after treatment initiation), blurred vision, diarrhea, back pain, headache, cough and vomiting. With reasonable data showing its long-term safety and disease improvement, fingolimod is a great alternative choice for patients with highly active RRMS and who prefer the oral treatment option.

#### 3.2.6. Mitoxantrone (Novantrone^®^, Immunex/Amgen, Thousand Oaks, CA, USA)

Mitoxantrone is primarily used to treat certain types of cancers, in particular, non-Hodgkin’s lymphoma, acute myeloid leukemia, breast and prostate cancer. Mitoxantrone is a type-II topoisomerase inhibitor, which disrupts DNA synthesis and DNA repair of cancer cells, however, normal cells are also affected. It is a potent immune suppressant, suppressing T cells, B cells and macrophages and reduces pro-inflammatory cytokines (IFN-γ, TNF-α, and IL-2) [138,139]. In patients with SPMS, intravenous injection of 12 mg/m^2^ mitoxantrone every 3 months up to 2 years resulted in reduced disability progression by 84% [140,141]. However, several side effects are associated with mitoxantrone which range from nausea, vomiting, hair loss, to, cardiotoxicity, leukemia, infertility, infection, leukopenia and thrombocytopenia [11]. As a result, its use has significantly been reduced over time. Furthermore, due to the risk of cardiotoxicity and leukemia, there is a limit on the cumulative lifetime dose to be administered to patients [11,142].

### 3.3. Treatment Using Humanized Monoclonal Antibodies

#### 3.3.1. Natalizumab (Tysabr^®^, Biogen, Cambridge, MA, USA)

Natalizumab is a humanized monoclonal antibody against the cellular adhesion molecule α4- integrin. Integrins are transmembrane receptors that enable cell-extracellular matrix adhesion activating cell signaling which regulate cell growth, division, survival, differentiation and migration. Integrins are expressed on T cells, B cells, monocytes, macrophages, NK cells, DC, neutrophils and eosinophils. Interfering or blocking α4-integrin affects immune cell migration across the blood brain barrier, thus, by blocking the interaction between α4-integrin and vascular endothelial adhesion molecule-1, inhibits transendothelial migration to the CNS [143]. Natalizumab is administered intravenously once a month [144] which reduces activated T cells within the CNS, resulting in anti-inflammatory responses and hence, neuroprotective effects [114]. In a phase III clinical trial natalizumab reduced brain lesions and the rate of disability progression up to 24 months [12,145]. In addition, natalizumab decreased by 92% of contrast-enhancing lesions, by 83% of new or expanding T2-weighted lesions, and by 76% in new T1-weighted hypointense lesions [146,147]. Natalizumab, was approved by the FDA in 2004, but was withdrawn due to 3 cases of rare brain infection, progressive multifocal leukoencephalopathy (PML; that usually leads to death or severe disability), but was re-introduced in 2006 under a special prescription program. However, by 2012 a further 212 cases (or 2.1/1000) of PML were reported to be attributed to natalizumab [148]. Despite these reports the FDA has not withdrawn natalizumab from the market as the clinical benefits outweigh the risks involved. Other side effects include, hepatotoxicity, allergic reactions and increased risks of infection. Due to the risks involved with natalizumab, there are reservations over its use as a preferred treatment option.

#### 3.3.2. Ofatumumab (Arzerra^®^, Novartis Pharma AG, Basel, Switzerland)

Ofatumumab (OMB157) is the first fully human type 1 IgG1 kappa (IgG1κ) monoclonal antibody and is currently licensed for the treatment (of patients with chronic lymphocytic leukemia (intravenously (iv), Arzerra^®^). It has also been shown to be beneficial to patients with rheumatoid arthritis, follicular non-Hodgkin’s lymphoma, diffuse B cell lymphoma and MS. B cells play a role in the pathogenesis of MS. B cells have essential functions in regulating immune response, by activating CD4^+^ T-cells and regulating T-cell responses via the secretion of cytokines and antibodies. B cells are present at demyelinating areas and in cerebrospinal fluid of patients with MS [149]. CD20 is a marker and present on the cell surface of all B cells. In an attempt to reduce the number of B cells including autoreactive B cells, the use of anti-CD20 antibodies would conceivably improve MS relapses and progression. In fact, there are several humanized anti-CD20 antibodies, such as rituximab [150], ocrelizumab [151] and ofatumumab [152], which have shown high efficacy in patients with RRMS. In 2015, Novartis acquired the rights from GlaxoSmithKline for the development of ofatumumab in oncology and other autoimmune indications. Ofatumumab binds to 2 unique novel epitopes on the CD20 molecule, induces B-cell depletion via complement dependent cytotoxicity and antibody-dependent cell-mediated cytotoxicity causing B cell apoptosis [153]. Ofatumumab has demonstrated high efficacy in hematologic malignancies and in rheumatoid arthritis. Based on 2 Phase II dosing human clinical studies, ofatumumab demonstrated high efficacy in reducing new MRI lesion activity more than 90% and was well tolerated in patients with MS [152]. Currently, ofatumumab is being further investigated in 2 Phase III trials (ASCLEPIOS I AND ASCLEPIOS II) and are recruiting patients with relapsing forms of MS (ofatumumab versus teriflunomide). The adaptive study design of both trials was recently presented by Hauser SL and colleagues at the American Academy of Neurology April 2017 in Boston, USA and results are highly anticipated [154].

#### 3.3.3. Ocrelizumab (Ocrevus^®^, Genentech Inc., San Fransisco, CA, USA)

A few months ago (March 2017), the FDA approved ocrelizumab to be used in PPMS, the first drug approved by the FDA for this form of MS and phase IV clinical trials were a requirement of the FDA to be conducted in order to determine the safety of ocrelizumab in younger patients with MS, ie, risk of cancer and effects on pregnancy (study outcomes due by 2024); although clinical trials in patients with lupus and rheumatoid arthritis were halted due to high rates of infections and increased risk of progressive multifocal leukoencephalopathy [155]. In addition, in patients with MS, there was an increased risk of breast cancer (6/781 females with MS on ocrelizumab compared to 0/668 females with MS in other trials) [155].

#### 3.3.4. Alemtuzumab (Lemtrada^®^, Sanofi Genzyme, Cambridge, MA, USA)

Alemtuzumab is a humanized monoclonal antibody against CD52, a cell surface molecule expressed on B and T cells; mature NK cells, plasma cells, neutrophils and importantly, hematological stem cells do not express CD52. In phase III clinical trials in patients with RRMS, alemtuzumab showed significantly lower annualized relapse rates and MRI measures (gadolinium-enhancing lesions, new or enlarging T2 lesions and brain atrophy) and were free of clinical disease longer, compared to IFNβ-1a [156,157]. Alemtuzumab can cause serious side effects including, immune thrombocytopenia, kidney problems, serious infusion problems (trouble breathing, swelling, chest pain, irregular heart beat), certain cancers (blood cancers, thyroid cancer), cytopenia and serious infections. It was approved by the FDA in 2014 to be used in RRMS patients, but due to the frequent and significant adverse events of alemtuzumab, it is generally used in patients with RRMS who have used 2 or more MS drugs and have failed to work.

#### 3.3.5. Daclizumab (Zinbryta^®^, Biogen, Cambridge, MA, USA)

Daclizumab is a humanized monoclonal antibody against CD25, the IL-2 receptor expressed on the surface of T cells. The mechanism by which daclizumab works is that it blocks the IL-2 receptor on T cells, preventing the activation of T cells. It was originally approved by the FDA in 1997 to prevent acute kidney transplants (together with corticosteroids and cyclosporine) however its use was halted due to low market demand. In recent years its use has re-emerged to treat patients with RRMS, it is injected subcutaneously, once a month [158]. In human clinical trials, daclizumab showed 45% reduced annualized relapse rates and 54% lower in the number of new lesions [158]. The side effects associated with daclizumab are relatively minor compared to other MS drugs, and include infections, skin rashes and liver complications.

## 4. New and Emerging Immunotherapeutic Strategies against MS

Antigen/peptide specific immunotherapy or using immune cells (i.e., stems cells), aim to restore tolerance while avoiding the use of non-specific immunosuppressive drugs as describe in Section 3, is a promising approach to fight autoimmune diseases including MS. As such, a number of approaches have been utilized.

### 4.1. Stem Cells

Multipotent hematopoietic stem cells (HSC) are cells isolated either from the bone marrow, umbilical cord blood or peripheral blood and are transplanted into the recipient. More commonly used for hematological malignancies (leukemia, multiple myeloma) its application has also expanded into autoimmune diseases. The first report of a bone marrow transplant in 1997 in a chronic myelogenous leukemia patient with MS which showed marked improvements in MS brain lesions [159] quickly led to the use of HSC transplantation (HSCT) in MS patients. HSCT in patients with active RRMS, reduce progression in about 70% of patients, decrease relapses dramatically and suppresses inflammatory MRI activity [160]. MS patients who have not responded to conventional therapy, who’s disease is aggressive with relapsing-remitting course and who are not presenting with high level of disability, are considered appropriate candidates for such treatment [161]. Although the clinical efficacy of HSCT long term has not been established. The mechanism by which HSCT works is that HSCT “reboots” the immune system and thus, prevents inflammation associated with the disease.

Mesenchymal stem cells (MSC) are isolated from an adult’s bone marrow, are differentiated in vitro for 2–3 weeks and re-injected back into the patient. In recent years a vast amount of research has been conducted in MSCs to treat MS with most studies being in mice and EAE models, and more recently in human clinical trials. In fact, in a pilot study in advanced MS patients, MSC transplantation improved expanded disability scale score with stabilization in 1/7 and disease progression in 1/7 patients and vision and low contrast sensitivity test showed improvement in 5/6 patients with 1/6 showing worsening effects [162]. In a phase II randomized double-blind, placebo-controlled crossover clinical trial showed lower mean cumulative number of lesions in patients receiving MSCs compared to placebo [163]. No serious adverse events were reported. The mechanism of action of MSC includes immunomodulation, neuroprotection and neuroregeneration [162]. The use of MSCs that reduce MRI parameters is a new and emerging research focus to develop new improved treatments for MS.

### 4.2. DNA Vaccine Studies

BHT-3009, a DNA vaccine that encodes the full-length human MBP, was developed with the aim to tolerize patients with MS against MBP [9,164,165]. In fact, in 30 patients with RRMS or SPMS who received 4 injections of BHT-3009 on weeks 1, 3, 5, 9 with escalating doses of 0.5 mg, 1.5 mg or 3 mg was reported to be safe and conferred positive changes on brain MRI and reduced the number of CD4^+^ T cells [9,164,166]. In addition, in a retrospective, randomized double blind, phase II study in 155 MS patients, BHT-3009 had no impact on the risk for persistent black holes (axonal loss and disability progression). However, there was a correlation to those who had generated high anti-IgM MBP antibodies to reduced risk of persistent black holes [167].

### 4.3. Nanoparticles

Nanoparticles have extensively been characterized and used as vaccine formulations in pre-clinical models of cancer and infectious diseases [168,169]. Polymeric biodegradable lactic-glycolic acid (PLGA) nanoparticles loaded with MOG_35–55_ peptide together with recombinant IL-10, were partially endocytozed by dendritic cells, secreted both MOG_35–55_ peptide and IL-10 in culture media for several weeks in vitro [170]. In mice, PLGA nanoparticles (MOG_35–55_ + IL-10) showed significant amelioration of EAE and reduction of IL-17 and IFN-gamma secretion by splenic T cells in vitro [170]. Recently, poly(ε-caprolactone) nanoparticles loaded with recombinant human MBP reduced IFN-gamma cytokines, reduced the clinical score and showed only mild histological changes of the myelin sheath [171]. Hence, nanoparticles as a delivery method of self-antigens are a promising tool to treat MS.

### 4.4. Altered Peptide Ligands

Altered peptide ligands (APL) are peptides closely related to the native (agonist) peptide with defined 1–2 substituted amino acid residues which interact with the T cell receptor (TCR) yet retains its binding ability to the MHC [65]. In phase I/II clinical trial by Neurocrine Biosciences Inc, used an APL of MBP_83−99_, where l-amino acids were changed to d-amino acids at positions 83, 84, 89, 91 (NBI-5788) [172]. However, this mode of APL induced T cell cross reactivity between the APL and the wild-type/agonist MBP_83−99_ peptide and adverse events in some patients resulted [173]. A subsequent multi-center double-blinded phase II clinical trial with NBI-5788 was suspended—Th2 responses were induced (IL-5, IL-13), however, 13/142 patients developed immediate-type hypersensitivity, who also generated anti-NBI-5788 antibodies which cross-reacted with native agonist MBP_83–99_ peptide [172,174]. National Institute of Neurological Disorders and Stroke sponsored trial, CGP77116, was used in a MRI-controlled phase II clinical trial. CGP77116, has Ala d-amino acids of MBP_83–99_ peptide at positions 83, 84, 89, 91 (CGP77116) of MBP_83–99_ peptide, in order to enhance stability [174]. However, this peptide was poorly tolerated at the dose tested, and the trial had to be discontinued. Three patients showed exacerbations to disease of which two were linked to CGP77116 injection with high IFN-gamma and low IL-4 (Th1-skewing) were secreted by activated CD4^+^ T cells. These CD4^+^ T cells also cross reacted with the native agonist MBP_83–99_ peptide [175]. Accordingly, the problems noted with both NBI-5788 and CGP77116 were likely due to inadequate pre-screening of APL effects on the many clonotypes against the targeted epitopes. Thus, although the APL was highly effective at blocking or switching some clones, it activated others. Thus, further pre-clinical testing is required and new modified peptides need to be designed, or a carrier needs to be used which further changes the resulting immune response.

#### 4.4.1. Cyclic Peptides 

Cyclization of peptides increases the stability, since linear peptides are sensitive to proteolytic enzymes. In addition, cyclic peptides are an important intermediate step and a useful template towards the rational design and development of non-peptide mimetics. While mimetic strategy is a challenging perspective it is worth pursuing in particular for MBP epitope-based MS therapy as it is still in its infancy. Efforts to design semi-mimetics of MBP_72–85_ epitope by combining non-natural amino acids as spacers and MBP epitope immunophores (Ser, Arg, Glu, Ala, Gln), led to substances that were effective to some extent in inducing the onset of EAE. Cyclic peptides are not only as a step towards non-peptide mimetics but also as putative therapeutics in MS [66].

Structure activity studies of the immunodominant agonist peptide MBP_87–99_, have shown that K^91^, P^96^ are important T cell receptor contact residues. Double mutation of K^91^, P^96^ to R^91^,A^96^ or single mutation of P^96^ to A^96^ (APL) of either in their linear or cyclic forms, results in suppression of EAE and decreased inflammation in the spinal cord of Lewis rats [71]. Single and double cyclic[A^91^]MBP_83–99_ peptide and cyclic[A^91^A^96^]MBP_83–99_ peptides emulsified in CFA induced IL-4 cytokines in SJL/J mice [62] however conjugation to reduced mannan further enhanced IL-4 cytokines with no IFN-gamma responses [63]. In guinea pigs and Lewis rats, cyclic[A^91^A^96^]MBP_83–99_ showed significantly reduced mechanical pain hypersensitivity compared to cyclic MBP_83–99_ peptide. This was associated with reduced T cell and macrophage infiltration to injured nerves of the spinal cord of animals [176,177,178]. In addition, these APL decreased CD4^+^ T cell line proliferation raised from a patient with MS, increased IL-10 cytokine secretion, bound to HLA-DR4 and were more stable to lysosomal enzymes (cathepsin B, D, H) compared to their linear counterparts [70]. Double mutation of K^91^, P^96^ to A^91^, A^96^ in either linear or cyclic forms were also shown to be active, with suppression of EAE in SJL/J mice, higher Th2 over Th1 cytokines produced, bound to HLA-DR4, the cyclic forms were more stable to lysosomal enzymes and induced high levels of IL-10 of peripheral blood mononuclear cells from patients with MS [61]. Recently, cyclic native agonist MOG_35–55_ peptide was shown to ameliorate clinical and neuropathological features of EAE in mice compared to its linear counterpart [179]. Thus, cyclic peptides, which offer greater stability and are able to modulate immune responses, are novel leads for the immunotherapy of many diseases, such as MS [66].

#### 4.4.2. Mannan as a Carrier to Modulate Immune Responses

Mannan, a polymannose, isolated from the wall of yeast cells has been shown to bind to the mannose receptor on dendritic cells as well as being a ligand for toll-like receptor 4 [180,181]. Mannan conjugated to MUC1 cancer protein induces immune responses in mice and protects mice against tumor challenge. This work was translated into human phase I, II and pilot III clinical trials; mannan-MUC1 induces protection against cancer recurrence at 18 years follow-up [182,183,184,185]. Furthermore, ex vivo cultured dendritic cells pulsed with mannan-MUC1 (CVac^TM^) and re-injection into patients induces strong cellular and clinical responses in ovarian cancer patients [186,187]. Due to the immunomodulatory properties of mannan, its effects as a carrier to MS peptides were determined.

Mutations of MBP_83–99_ agonist native peptide to result in mutant peptides (APL)—linear [A^91^]MBP_83–99_, [E^91^]MBP_83–99_, [F^91^]MBP_83–99_, [Y^91^]MBP_83–99_ and [R^91^, A^96^]MBP_83–99_, induced IFN-gamma albeit reduced compared to the native agonist peptide, however, only the double APL [R^91^, A^96^]MBP_83–99_ induced IL-4 secretion by T cells and antagonized IFN-gamma production in vitro by T cells against the native MBP_83–99_ peptide [67]. In addition, T cells against the native MBP_83–99_ peptide cross-reacted with all peptides except [Y^91^]MBP_83–99_ and [R^91^,A^96^]MBP_83–99_ [68]. Conjugation of [R^91^,A^96^]MBP_83–99_, [A^91^,A^96^]MBP_83–99_, F^91^]MBP_83–99_, [Y^91^]MBP_83–99_ peptides to mannan, completed abrogated IFN-gamma responses and elicited high IL-4 (i.e., Th1 to Th2 switch) [63,69,188]. Likewise, linear double-mutant APL [L^144^R^147^]PLP_139–151_ induces high levels of IL-4, and cyclization of this analog elicited low levels of IFN-gamma. When conjugated to mannan, [L^144^R^147^]PLP_139–151_ peptide completely abrogated IFN-gamma, while both linear and cyclic native agonist PLP_139–151_ peptides stimulated IFN-gamma secreting T cells [64]. Furthermore, mannan conjugated to the immunodominant agonist MOG_35–55_ peptide primes non-pathogenic Th1 and Th17 cells and ameliorates EAE in mice [73]; a phase I human clinical trial is planned using mannan conjugated to MOG_35–55_ peptide later this year. It is clear that, mannan is able to divert immune responses from Th1 to Th2 and is a promising carrier for further studies for the development of immunotherapeutics against MS.

## 5. Symptomatic Medication

### Dalfampridine (Ampyra/Fampyra^®^, Acorda Therapeutics)

Dalfampridine is not intended to delay symptoms or change the course of disease, but rather, to improve motor symptoms such as walking. Dalfampridine, is a potassium channel blocker, resulting in improved potassium currents and nerve conductance. Dalfampridine is used in patients who have had MS for more than 3 years and it was approved by the FDA in 2010. Common side effects include nausea, nervousness and dizziness, which are relatively minor compared to other MS drugs.

## 6. Conclusions and Future Prospects

MS is an autoimmune disorder of the CNS with an array of immune cells being either activated or suppressed leading to demyelination and disease progression. In addition, genetic predisposition, viral mimicry, vitamin and mineral deficiency, geographical location are also etiological factors that contribute to disease. More recently, citrulination of myelin peptides have been shown to contribute to disease activation [59,60]. A number of treatment options are available to patients with MS, in particular those with active disease, however due to side effects, limited long term effectiveness and inability to reverse disease, new improved treatment options are required. As described here a number of new and upcoming promising therapeutic candidates are becoming available, although their effectiveness in human clinical trials remains to be determined. Recently, it was reported that non-peptide mimetics mapping the MBP_83–96_ T cell epitope can function as T cell receptor antagonists, hence such an approach may pave the way to developing alternative and improved immunotherapeutics against MS [189]. With the plethora of information regarding the immunopathophysiology of MS and availability of treatment options and new upcoming treatments, the future holds promise for managing and treating the disease.

## Figures and Tables

**Table 1:** Disease-modifying drugs available to patients with RRMS.

Drug	Brand	Dose	Number of of Injections, Route	Actions
IFN-β1a	Avonex^®^	7.5 mg 1st dose	1/week, i.m	Balances pro- and anti-inflammatory cytokines
		15 mg 2nd dose		Decreases Th17 cells
		22.5 mg 3rd dose		Decreases IL-17
		30 mg all subsequent doses		
	Rebif^®^	22 mg or 44 mg	3/week, s.c	
IFN-β1b	Betaseron^®^	62.5 mg and increase over 6 weeks to 250 mg	1/2 days, s.c	
	Extavia^®^	62.5 mg and increase over 6 weeks to 250 mg	1/2 days, s.c	
pegIFN-β1a	Plegridy^®^	63 mg 1st dose	1/2 weeks, s.c	
		95 mg 2nd dose		
		125 mg all subsequent doses		
Glatiramer acetate, EKAY	Copaxone^®^	20 mg or 40 mg	1/day, s.c	Blocks pMHC
			3/week, s.c	
Dimethyl fumarate	Tecfidera^®^	240 mg	2–3/day, oral	Anti-inflammatory Anti-oxidative stress
Teriflunomide	Aubagio^®^	7 or 14 mg	1/day, oral	Inhibits dihydroorotate dehydrogenase, T, B cells and IFN-γ secreting T cells
Fingolimod	Glenya^®^	0.5 mg	1/day, oral	Antagonist of SIP receptor Decrease T, B cells activates SIP signaling in CNS
Mitoxantrone	Novatrone^®^	12 mg/m^2^	1/3 months up to 2 years	Suppresses T, B cells and macrophages. Reduces Th1 cytokines
Dalfampridine	Ampyra^®^	10 mg	2/day, oral	Potassium channel blocker Improves motor symptoms, i.e., walking
**Humanized Monoclonal Antibody Treatments**				
Natalizumab	Tysabr^®^	300 mg	1/28 days, i.v	Humanized anti-α4-integrin Mab. Affects cell migration, division, growth and survival
Ofatumumab	Arzerra^®^	3–700 mg	1/2 weeks, i.v	Humanized anti-CD20 Mab. Cytotoxic to CD20+ cells via CDC and ADCC
Ocrelizumab	Ocrevus^®^	300–600 mg	300 mg weeks 1 and 3, then 600 mg 1/6 months, i.v	Humanized anti-CD20 Mab
Alemtuzumab	Lemtrada^®^	12 mg	5 days in a row; after 1 year, 3 days	Humanized anti-CD52 Mab. Depletes T, B cells, increases Treg, Th2, decrease Th1 cells
Daclizumab	Zinbryta^®^	150 mg	1/month, s.c	Humanized anti-CD25 Mab.Blocks IL-2R, decreases T cells, increases NK cells

ADCC, antibody-dependent cellular cytotoxicity; CDC, complement-dependent cytotoxicity; DC, dendritic cells; EKAY, single amino acid code for l-glutamic acid, lysine, alanine, tyrosine; IFN, interferon; IL-2R, interleukin-2 receptor; i.m, intramuscular; i.v, intravenous; Mab, monoclonal antibodies; NK, natural killer cells; pegIFN, polyethylene glycol linked to IFN; pMHC, peptide-major histocompatibility complex; RRMS, relapsing remitting multiple sclerosis; s.c, subcutaneous; SIP, sphingosine-1-phosphate; Th, helper T cells; Treg, regulatory T cells (CD4^+^CD25^+^FoxP3^+^).
